# Impact of Rosuvastatin Treatment on HDL-Induced PKC-*β*II and eNOS Phosphorylation in Endothelial Cells and Its Relation to Flow-Mediated Dilatation in Patients with Chronic Heart Failure

**DOI:** 10.1155/2016/4826102

**Published:** 2016-08-02

**Authors:** Ephraim B. Winzer, Pauline Gaida, Robert Höllriegel, Tina Fischer, Axel Linke, Gerhard Schuler, Volker Adams, Sandra Erbs

**Affiliations:** ^1^Leipzig Heart Center, Department of Cardiology, Leipzig University, 04289 Leipzig, Germany; ^2^Saechsisches Krankenhaus Altscherbitz, 04435 Leipzig, Germany

## Abstract

*Background*. Endothelial function is impaired in chronic heart failure (CHF). Statins upregulate endothelial NO synthase (eNOS) and improve endothelial function. Recent studies demonstrated that HDL stimulates NO production due to eNOS phosphorylation at Ser^1177^, dephosphorylation at Thr^495^, and diminished phosphorylation of PKC-*β*II at Ser^660^. The aim of this study was to elucidate the impact of rosuvastatin on HDL mediated eNOS and PKC-*β*II phosphorylation and its relation to endothelial function.* Methods*. 18 CHF patients were randomized to 12 weeks of rosuvastatin or placebo. At baseline, 12 weeks, and 4 weeks after treatment cessation we determined lipid levels and isolated HDL. Human aortic endothelial cells (HAEC) were incubated with isolated HDL and phosphorylation of eNOS and PKC-*β*II was evaluated. Flow-mediated dilatation (FMD) was measured at the radial artery.* Results*. Rosuvastatin improved FMD significantly. This effect was blunted after treatment cessation. LDL plasma levels were reduced after rosuvastatin treatment whereas drug withdrawal resulted in significant increase. HDL levels remained unaffected. Incubation of HAEC with HDL had no impact on phosphorylation of eNOS or PKC-*β*II.* Conclusion*. HDL mediated eNOS and PKC-*β*II phosphorylation levels in endothelial cells do not change with rosuvastatin in CHF patients and do not mediate the marked improvement in endothelial function.

## 1. Introduction

Patients with chronic heart failure (CHF) are characterized by endothelial dysfunction which is associated with worse prognosis [1]. Different mechanisms have been shown to contribute to endothelial dysfunction. A hallmark is an imbalance of nitric oxide (NO) production via the endothelial NO synthase (eNOS) and NO degradation via oxidant radicals resulting in a diminished NO bioavailability [2, 3].

High-density lipoprotein (HDL) plasma levels are of prognostic relevance for cardiovascular diseases. Nevertheless, several pharmacological approaches to increase HDL quantity failed to reduce cardiovascular events [4]. Therefore, functional properties of HDL are of increasing interest [5]. HDL from healthy subjects has been shown to activate NO synthesis in endothelial cells [6, 7]. In vitro experiments demonstrated that the incubation of murine aortic rings with human HDL induces NO dependent vasorelaxation [6]. The role of HDL in endothelial function is underscored by in vivo studies showing that intravenous infusion of reconstituted HDL in hypercholesterolemic men rapidly restores endothelial function by increasing NO bioavailability [8]. Furthermore, endothelial function in children was found to be correlated to the ability of isolated HDL to induce eNOS activating phosphorylation in cultured endothelial cells [9].

In CHF, functional properties of HDL are altered. With increasing disease severity, more malondialdehyde is bound to HDL particles and subsequent activation of protein kinase-*β*II (PKC-*β*II) results in pronounced phosphorylation of eNOS at its inhibitory site Thr^495^ whereas the eNOS activating phosphorylation at Ser^1177^, mediated by PI3K and Akt, is reduced. Thereby, the stimulating effect of isolated HDL on NO production in endothelial cells is blunted. However, this process seems partially reversible with exercise training [7].

Numerous studies have shown that statins improve endothelial function mediated by increased eNOS expression, reduced oxidant stress, and restored endothelial repair mechanisms via circulating endothelial progenitor cells [10–12].

Recently, Chang et al. found that HDL isolated from patients with valvular heart disease in comparison to healthy controls inhibited phosphorylation of eNOS at Ser^1177^ and increased phosphorylation at Thr^495^ in cultured endothelial cells. This was associated with reduced eNOS-dependent NO production and increased superoxide generation and resulted in impaired vasodilation of murine aortic rings in response to acetylcholine. Interestingly, a short time treatment with simvastatin partially corrected these dysfunctional properties of HDL [13].

These findings indicate that the statin related improvement in endothelial function in humans might be partially mediated by functional properties of HDL.

Previously, we reported the effects of a high-dose rosuvastatin therapy for 12 weeks in patients with CHF on flow-mediated dilatation (FMD) of the radial artery and endothelial repair mechanisms [11]. To further elucidate the role of HDL in this context, the aim of the present study was to analyse the effect of isolated HDL on the phosphorylation of PKC-*β*II and eNOS at Ser^1177^ and Thr^495^ in cultured endothelial cells. Furthermore, we report for the first time the effect of statin withdrawal on blood lipids and endothelial function in CHF patients.

## 2. Methods

The main clinical trial is registered at https://clinicaltrials.gov/ with the following number: NCT00176332.

### 2.1. Patient Selection and Study Protocol

The study protocol was approved by the ethics committee of the University of Leipzig and written informed consent was obtained from all patients.

In a subgroup of 18 patients from the main study cohort, further evaluation of HDL function was performed (rosuvastatin group *n* = 9, placebo group *n* = 9). Patients with CHF as a result of either ischemic heart disease or dilated cardiomyopathy, clinically stable at New York Heart Association class II or class III for at least one month, left ventricular-ejection fraction <40%, end diastolic left ventricular diameter >55 mm, and peak oxygen uptake <20 mL/min/kg body weight were included. Significant valvular disease and ongoing nicotine abuse served as exclusion criterions. Patients were randomly assigned in a double blind manner to an intervention group receiving 40 mg rosuvastatin daily or a placebo group. At the beginning of the study (bsl), after 12 weeks of active treatment (12 wk), and 4 weeks after discontinuation of the study drug (16 wk), blood samples were taken from all patients to determine blood lipid levels and isolate HDL particles.

### 2.2. Endothelial Function Measurement

Flow-mediated dilatation (FMD) of the radial artery was measured using a high-resolution ultrasound scanning echo-tracking angiometer (NIUS 02, Asulab Research Laboratory, Neuchatel, Switzerland) as described previously [11].

### 2.3. Isolation of HDL

HDL was isolated from serum by sequential density ultracentrifugation (*d* = 1.006–1.21 g/mL) as recently described in detail [7].

### 2.4. Cell Culture and Incubation with Isolated HDL

Human aortic ECs (HAEC; Cell Systems Biotechnology, Troisdorf, Germany) were cultured in EGM-2 cell culture medium (Lonza, Walkersville, MD): Cells were incubated for 0, 5, 10, 15, 30, or 60 minutes with 50 *μ*g/mL isolated HDL in EGM-2 medium (containing growth factors and 10% fetal calf serum) as recently described [7, 14, 15]. Thereafter, cells were harvested with ice-cold lysis buffer (50 mmol/L Tris-HCl; pH 7.4; 1% NP-40; 0,25% Na-deoxycholate; 150 mmol/L NaCl; 1 mmol/L EDTA; 0.1% Triton X-100; 0.2% SDS) containing protease inhibitor mix M (Serva, Heidelberg, Germany) as well as phosphatase inhibitor mix II (Serva). Protein concentration was determined using BSA as standard (BCA method; Pierce, Rockford, IL).

### 2.5. Western Blot Analysis

Ten micrograms of total protein was separated on a denaturing polyacrylamide gel and transferred to a PVDF membrane. To detect specific proteins, the following antibodies were applied: anti-eNOS (Santa Cruz), antiphospho-eNOS-Ser^1177^ and antiphospho-eNOS-Thr^495^ (both BD Biosciences, Heidelberg, Germany), and anti-PKC-*β*II and antiphospho-PKC-*β*II-Ser^660^ (both Santa Cruz). For the evaluation of HDL-induced phosphorylation of the respective protein, the maximal stimulation was used as recently described [7]. All samples were analysed in triplicate.

### 2.6. Statistical Analysis

Data were analysed using SPSS version 22 (IBM Corp.). For the descriptive statistics of clinical parameters, median and interquartile range were calculated. Mean values ± standard error (SEM) was calculated for all other variables. Repeated measures ANOVA were used to test for change over time. Comparisons from baseline were performed using an analysis of covariance. Categorical variables were tested applying Fisher's exact test. Correlation between selected variables was estimated by Spearman's rank correlation coefficient. A *p* value of less than 0.05 was considered statistically significant.

## 3. Results

### 3.1. Study Patients

The characteristics of the study patients are depicted in Table 1. The randomly assigned patients for HDL function analysis represent well the whole study group of the main study (data not shown) [11]. Clinical characteristics of the patients and cardiac medication did not differ between groups.

### 3.2. Blood Lipid Levels

LDL plasma levels were reduced after 12 weeks of rosuvastatin treatment, whereas drug withdrawal resulted in a significant LDL increase (bsl 3.30 ± 0.17 mmol/L, 12 wk 1.53 ± 0.09 mmol/L, and 16 wk 3.49 ± 0.19 mmol/L; *p* < 0.001 for bsl versus 12 wk and 12 wk versus 16 wk). However, HDL levels remained unaffected (bsl 1.17 ± 0.10 mmol/L, 12 wk 1.26 ± 0.08 mmol/L, and 16 wk 1.16 ± 0.09 mmol/L; *p* = n.s.) (Table 2).

### 3.3. Endothelial Function

As already shown in an earlier publication, the flow-mediated dilatation of the radial artery significantly improved by 183% with rosuvastatin treatment without any change in the placebo group [11]. The FMD data of the presented subgroup here are depicted in Table 2. The treatment effect did not persist four weeks after drug withdrawal.

### 3.4. Phosphorylation of Endothelial NO Synthase and Protein Kinase C-*β*II

**Figure 1:**
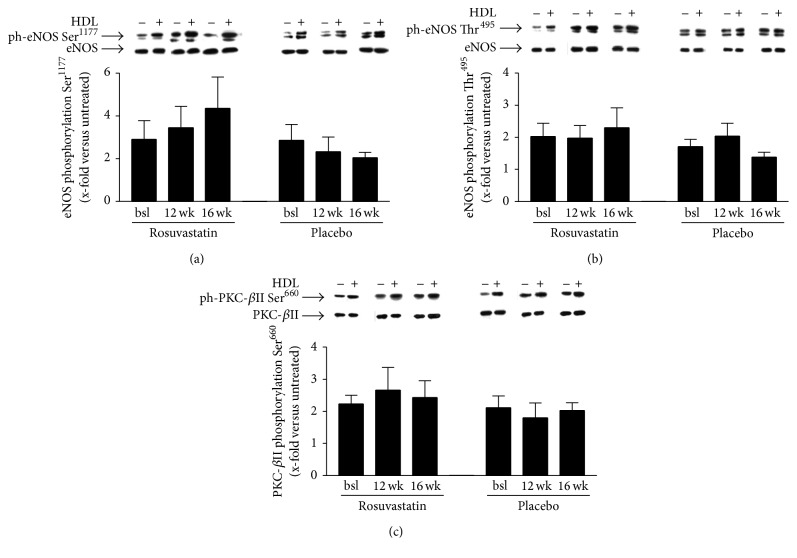
(a) X-fold increase in eNOS phosphorylation at Ser^1177^ of human aortic endothelial cells stimulated with HDL from patients treated with rosuvastatin or placebo versus unstimulated cells. (b) X-fold increase in eNOS phosphorylation at Thr^495^ of human aortic endothelial cells stimulated with HDL from patients treated with rosuvastatin or placebo versus unstimulated cells. (c) X-fold increase in PKC-*β*II phosphorylation at Ser^660^ of human aortic endothelial cells stimulated with HDL from patients treated with rosuvastatin or placebo versus unstimulated cells.

Incubation of HAEC with HDL from rosuvastatin treated patients had no impact on phosphorylation of eNOS at Ser^1177^ (bsl 2.91 ± 0.86-fold, 12 wk 3.45 ± 1.0-fold, and 16 wk 4.35 ± 1.47-fold versus unstimulated cells; *p* = n.s.) or Thr^495^ (bsl 2.03 ± 0.41-fold, 12 wk 1.98 ± 0.39-fold, and 16 wk 2.30 ± 0.62-fold versus unstimulated cells; *p* = n.s.) and does not influence phosphorylation of PKC-*β*II at Ser^660^ (bsl 2.23 ± 0.27-fold, 12 wk 2.66 ± 0.71-fold, and 16 wk 2.43 ± 0.53-fold versus unstimulated cells; *p* = n.s.). In the placebo group, no change in these parameters was evident (Figure 1).

### 3.5. Correlation between Endothelial Function and Phosphorylation of eNOS

In the rosuvastatin group, we found no correlation between flow-mediated dilatation and phosphorylation of eNOS neither at Ser^1177^ at any time point (bsl *r* = −0.65, *p* = 0.06; 12 wk *r* = 0.15, *p* = 0.70; 16 wk *r* = 0.15, *p* = 0.70) nor at Thr^495^ (bsl *r* = 0.20, *p* = 0.61; 12 wk *r* = −0.13, *p* = 0.73; 16 wk *r* = 0.63, *p* = 0.07) or PKC-*β*II at Ser^660^ (bsl *r* = −0.18, *p* = 0.64; 12 wk *r* = −0.17, *p* = 0.67; 16 wk *r* = −0.40, *p* = 0.29).

## 4. Discussion

The following findings emerge from this study: (i) Treatment with rosuvastatin 40 mg for 12 weeks in patients with chronic heart failure does not affect the ability of isolated HDL to change the phosphorylation of PKC-*β*II at Ser^660^ and the phosphorylation of eNOS at its activity regulating sites Ser^1177^ and Thr^495^ in cultured endothelial cells. (ii) Four weeks after rosuvastatin withdrawal, the treatment effect on LDL cholesterol level and endothelial function is completely abolished.

Our data are in contrast to findings by Chang et al. who demonstrated that low-dose simvastatin treatment with 20 mg per day even for the short period of 5–7 days in patients with valvular heart disease was sufficient to induce HDL-mediated phosphorylation of Akt and eNOS at Ser^1177^ and to inhibit eNOS phosphorylation at the Thr^495^ site in cultured human umbilical vein endothelial cells, resulting in enhanced NO production. Vasodilation of murine aortic rings significantly increased when incubated with HDL from simvastatin treated patients compared to HDL from untreated patients in this study [13].

Beside different statins used with different dosages and treatment periods, the patient groups studied differ significantly. We selected heart failure patients with reduced left ventricular-ejection fraction (mean LV-EF 30% compared to 62% in the study by Chang et al.) and excluded those with more than mild-to-moderate valvular disease. Additionally, more than one-third of patients in our group suffered from ischemic heart disease due to coronary artery disease whereas Chang et al. excluded patients with coronary artery disease. A previous study from our group indicated that HDL dysfunction in patients with reduced LV-EF is related to disease severity with lower HDL-induced NO production in patients according to New York Heart Association Classification class IIIb compared to those in class II [7]. Interestingly, Chang et al. did not find a relation between heart failure symptoms caused by valvular disease and HDL function with a higher proinflammation index in patients in NYHA class II than those in class III. This raises the question whether different mechanisms result in HDL dysfunction in patients with ischemic or dilative cardiomyopathy compared to those with valvular heart disease with subsequently different responsiveness to statin treatment.

The amount of malondialdehyde (MDA) bound to HDL particles has been shown to be crucial for HDL-mediated activation of PKC-*β*II and downstream eNOS phosphorylation at Thr^495^ resulting in diminished NO production and endothelial dysfunction [7, 16]. Paraoxonase (Pon), an enzyme associated with HDL, protects lipoproteins from oxidative modifications. In CHF, Pon activity of HDL is significantly reduced when compared to healthy controls and might thereby contribute to reduced HDL function in CHF [7]. Also in coronary artery disease patients, HDL associated Pon activity was found to be reduced and associated with higher MDA levels bound to HDL and the inability of isolated HDL to induce NO production in cultured endothelial cells [17]. It has been shown that simvastatin as well as atorvastatin significantly increases serum Pon activity in association with reduced lipid peroxide concentration and serum levels of MDA in hypercholesterolemia [18, 19]. In the present study, PKC-*β*II phosphorylation remained totally unchanged in response to rosuvastatin even though serum markers of oxidant stress including oxidized LDL and lipid peroxide concentration were reduced as already reported [11]. We therefore assume that rosuvastatin does not reduce the level of MDA bound to HDL particles significantly in CHF. We speculate that the previously demonstrated changes in serum Pon activity and MDA level with statin therapy do not necessarily reflect the activity of Pon and the amount of MDA associated with HDL particles. Nevertheless, differences in patient population as well as type of statin and dosage might contribute to these conflicting results.

However, results from cell culture experiments indicate that statin treatment might improve HDL-mediated eNOS activity via changes on the receptor site: Apolipoprotein A1, a key lipoprotein in HDL particles, binds to the scavenger receptor-BI on endothelial cells [6]. The stimulation of human umbilical vein endothelial cells with HDL resulted in an SR-BI dependent upregulation and activation of eNOS. Prior treatment of these cells with simvastatin increased SR-BI expression through a RhoA and PPAR-*α* dependent mechanism and thereby enhanced downstream effects on eNOS [20].

Additionally, another signalling pathway evolved in HDL-mediated eNOS activation was found to be modified by statins: sphingosine 1-phosphate (S1P), which is enriched in HDL fractions, binds to G-protein-coupled S1P receptors on endothelial cells and regulates eNOS activation [21]. The incubation of bovine aortic endothelial cells with pitavastatin or atorvastatin led to a dose-dependent increase in S1P1-receptor expression and enhanced eNOS activation in response to HDL [22]. Both of these signalling pathways regulate eNOS activity via phosphorylation at Ser^1177^ in a PI3K/Akt-dependent manner [16, 23, 24].

To demonstrate such treatment effects in humans, endothelial cells would have to be harvested before and after oral statin treatment. This has not been performed in the present study.

Recent analysis of the West of Scotland Coronary Prevention Study identified a long-term legacy benefit from five years of cholesterol lowering therapy with pravastatin in middle aged men at increased risk for atherosclerosis through decreased cardiovascular event rates and death from cardiovascular causes even 15 years after the active treatment period [25]. However, in our trial, LDL cholesterol level as well as FMD reached baseline levels four weeks after rosuvastatin withdrawal. This is in line with findings in patients with metabolic syndrome showing that the discontinuation of fluvastatin results in deterioration of FMD already after 24 hours [26]. This might be of clinical relevance since an early rebound effect of endothelial dysfunction following acute statin withdrawal is discussed to be associated with worse prognosis at least in patients with acute vascular stress such as stroke or acute coronary syndrome [27].

There are some limitations: First, the sample size for the analysis of HDL function is quite low. However, we found not even a trend towards altered eNOS phosphorylation levels after rosuvastatin therapy, whereas lipid levels and endothelial function clearly changed with therapy and drug withdrawal. Second, we did not evaluate the effect of rosuvastatin on SR-BI or S1P receptor expression and Pon activity and MDA. Nevertheless, primary goal of the study was to investigate the influence of rosuvastatin on eNOS activity. Third, NO generation in endothelial cells in response to HDL was not quantified, and we cannot rule out the possibility that rosuvastatin ameliorates NO generation independent of the evaluated phosphorylation levels.

## 5. Conclusion

Rosuvastatin treatment in CHF patients does not alter the ability of isolated HDL on the phosphorylation levels of PKC-*β*II and eNOS at Ser^1177^ and Thr^495^ in cultured endothelial cells. We did not find any relation between FMD and HDL-induced PKC-*β*II and eNOS phosphorylation with or without rosuvastatin treatment. This indicates that HDL function, at least its effect on eNOS phosphorylation, does not mediate the marked improvement in endothelial function with rosuvastatin treatment in CHF patients.

## Figures and Tables

**Table 1:** Clinical characteristics.

	Rosuvastatin *n* = 9	Placebo *n* = 9	*p* value
*Clinical profile*			
Age [years]	67 (57–72)	60 (55–73)	0.65
Male gender [*n*]	7 (78%)	5 (56%)	0.62
*Characterization of CHF*			
Ischemic heart disease [*n*]	3 (33%)	4 (44%)	1.00
LV ejection fraction [%]	34 (24–36)	30 (30–37)	0.37
LV end diastolic diameter [mm]	64 (58–71)	59 (58–65)	0.19
VO_2_max [mL/min/kg]	13.7 (11.5–17.0)	16.7 (13.8–19.1)	0.18
NYHA class II/III [*n*/*n*]	4/5 (44/56%)	6/3 (67/33%)	0.64
*Cardiac medication*			
Beta blocker [*n*]	9 (100%)	9 (100%)	n.a.
ACE inhibitor or AT II blocker [*n*]	9 (100%)	9 (100%)	n.a.
Aldosterone antagonist [*n*]	7 (78%)	6 (67%)	1.00
Other diuretics [*n*]	8 (89%)	8 (89%)	1.00
Digitalis [*n*]	1 (11%)	3 (33%)	0.24

n.a.: not applicable, median (IQR).

**Table 2:** Flow-mediated dilatation and blood lipids.

	Baseline	12 weeks	16 weeks
FMD [%]			
* Rosuvastatin*	7.56 ± 1.61	21.36 ± 3.49^*∗*^	6.23 ± 1.16^*∗∗*^
* Placebo*	4.73 ± 0.88	4.74 ± 1.01	5.14 ± 0.68
LDL [mmol/L]			
* Rosuvastatin*	3.30 ± 0.17	1.53 ± 0.09^*∗*^	3.49 ± 0.19^*∗∗*^
* Placebo*	3.91 ± 0.27	3.12 ± 0.36	3.31 ± 0.45
HDL [mmol/L]			
* Rosuvastatin*	1.17 ± 0.10	1.26 ± 0.08	1.16 ± 0.09
* Placebo*	1.21 ± 0.12	1.19 ± 0.10	1.28 ± 0.14

^*∗*^Repeated measures ANOVA *p* < 0.01 for baseline versus 12 weeks.

^*∗∗*^Repeated measures ANOVA *p* < 0.01 for 12 weeks versus 16 weeks.

## Abbreviations

Akt: Protein kinase B
CHF: Chronic heart failure
eNOS: Endothelial nitric oxide synthase
FMD: flow-mediated dilatation
HAEC: Human aortic endothelial cell
HDL: High-density lipoprotein
LDL: Low-density lipoprotein
LV-EF: Left ventricular-ejection fraction
MDA: Malondialdehyde
NO: Nitric oxide
PI3K: Phosphatidyl inositol 3 phosphate kinase
PKC: Protein kinase C
Pon: Paraoxonase
S1P: Sphingosine 1 phosphate
ser: Serine
SR-BI: Scavenger receptor-BI
thr: Threonine.
